# Initial Feasibility of the “Families Moving Forward Connect” Mobile Health Intervention for Caregivers of Children With Fetal Alcohol Spectrum Disorders: Mixed Method Evaluation Within a Systematic User-Centered Design Approach

**DOI:** 10.2196/29687

**Published:** 2021-12-02

**Authors:** Christie Lynn McGee Petrenko, Carson Christine Kautz-Turnbull, Alicia Rose Roth, Jennifer Elizabeth Parr, Cristiano Tapparello, Utku Demir, Heather Carmichael Olson

**Affiliations:** 1 Mt. Hope Family Center University of Rochester Rochester, NY United States; 2 Department of Electrical and Computer Engineering University of Rochester Rochester, NY United States; 3 Department of Psychiatry and Behavioral Sciences University of Washington School of Medicine Seattle, WA United States; 4 Center for Child Health, Behavior, and Development Seattle Children’s Research Institute Seattle, WA United States

**Keywords:** fetal alcohol spectrum disorders, fetal alcohol syndrome, intervention, mobile health, mHealth, parenting, children, prenatal alcohol, digital health, user-centered design, mobile phone

## Abstract

**Background:**

Fetal alcohol spectrum disorders (FASD) are prevalent neurodevelopmental conditions. Significant barriers prevent family access to FASD-informed care. To improve accessibility, a scalable mobile health intervention for caregivers of children with FASD is under development. The app, called Families Moving Forward (FMF) Connect, is derived from the FMF Program, a parenting intervention tailored for FASD. FMF Connect has 5 components: Learning Modules, Family Forum, Library, Notebook, and Dashboard.

**Objective:**

This study assesses the feasibility of FMF Connect intervention prototypes. This includes examining app usage data and evaluating user experience to guide further refinements.

**Methods:**

Two rounds of beta-testing were conducted as part of a systematic approach to the development and evaluation of FMF Connect: (1) an iOS prototype was tested with 20 caregivers of children (aged 3-17 years) with FASD and 17 providers for the first round (April-May 2019) and (2) iOS and Android prototypes were tested with 25 caregivers and 1 provider for the second round (November-December 2019). After each 6-week trial, focus groups or individual interviews were completed. Usage analytics and thematic analysis were used to address feasibility objectives.

**Results:**

Across beta-test trials, 84% (38/45) of caregivers and 94% (17/18) of providers installed the FMF Connect app. Technological issues were tracked in real time with updates to address problems and expand app functionalities. On use days, caregivers averaged 20 minutes using the app; most of the time was spent watching videos in Learning Modules. Caregiver engagement with the Learning Modules varied across 5 usage pattern tiers. Overall, 67% (30/45) of caregivers posted at least once in the Family Forum. Interviews were completed by 26 caregivers and 16 providers. App evaluations generally did not differ according to usage pattern tier or demographic characteristics. Globally, app users were very positive, with 2.5 times more positive- than negative-coded segments across participants. Positive evaluations emphasized the benefits of accessible information and practical utility of the app. Informational and video content were described as especially valuable to caregivers. A number of affective and social benefits of the app were identified, aligning well with the caregivers’ stated motivators for app use. Negative evaluations of user experience generally emphasized technical and navigational aspects. Refinements were made on the basis of feedback during the first beta test, which were positively received during the second round. Participants offered many valuable recommendations for continuing app refinement, which is useful in improving user experience.

**Conclusions:**

The results demonstrate that the FMF Connect intervention is acceptable and feasible for caregivers raising children with FASD. They will guide subsequent app refinement before large-scale randomized testing. This study used a systematic, user-centered design approach for app development and evaluation. The approach used here may illustrate a model that can broadly inform the development of mobile health and digital parenting interventions.

## Introduction

### Background

Fetal alcohol spectrum disorders (FASD) are a range of conditions associated with prenatal alcohol exposure (PAE) [1]. PAE can affect the development of the brain and other organ systems, resulting in neurodevelopmental impairment and high rates of physical and mental health problems [2,3]. FASD are highly prevalent, occurring in an estimated 1%-5% of the US population [4]. Unfortunately, FASD are markedly underrecognized, and most individuals do not receive an accurate diagnosis [4,5]. Families often only know an individual was exposed to PAE and is showing learning or behavior concerns. Many barriers to care exist because of the pervasive lack of knowledge about FASD across service systems and in the broader community [6,7].

People with FASD have important strengths, including social motivation, resilience, and individual passions and talents [8,9]. They strive to be included and contribute meaningfully to their communities. Caregivers (ie, biological, foster, adoptive, and relatives) are dedicated to supporting their children with FASD or PAE and undertake numerous protective actions to reduce system barriers and help their own children and families adapt to challenges [10-12]. However, responding to FASD remains a very stressful experience, often fraught with difficulties accessing the resources and information needed. A growing number of evidence-based interventions have been studied in preschool and school-aged children with FASD [13,14]. It is unfortunate that none of these are, as yet, widely available in community settings. Thus, innovative and scalable solutions are required.

### A Systematic, User-Centered Design Approach to App Development and Evaluation: The Example of Families Moving Forward Connect Programmatic Research

To address significant barriers to care affecting this population, we developed a mobile health (mHealth) intervention called Families Moving Forward (FMF) Connect. mHealth, or the application of smartphones or wireless technologies to improve health, has bourgeoned since the emergence of app stores in 2008 [15]. mHealth has many potential advantages, including increasing health care capacity, providing patient access to tailored and immediate support, reducing stigma in obtaining care, and improving cost-effectiveness [16]. FMF Connect is the first known mHealth app developed and tested for FASD.

**Figure 1:**
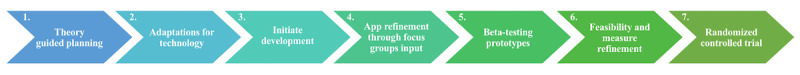
Phases of a systematic, user-centered design approach to mobile health intervention development and evaluation.

The task of developing and evaluating the FMF Connect mHealth intervention is being carried out following a systematic, user-centered design approach to app development and evaluation (Figure 1). Unfortunately, deployment of this type of systematic approach has been relatively rare for mHealth interventions [17-19]. This methodology integrates user-centered design principles, which emphasize understanding users, tasks, and environments, with the process of obtaining iterative and collaborative input from users [20-22]. There are seven phases to this approach, as operationalized in Figure 1. Of course, the process is more complex than that illustrated in Figure 1. There are certainly feedback loops between phases that indicate iterative change. This study describes the model, which involves a multidisciplinary development team and engagement of key stakeholders through focus groups and beta-testing. We also include the presentation of data from the fifth phase of the model to reveal specifically how user data from beta-testing can strategically refine and enhance app design. We note that this generalized approach can be used in the broader field of mHealth development.

In the first phase of this approach, the self-directed FMF Connect app was carefully derived from the empirically supported, therapist-led FMF Program developed by Heather Carmichael Olson, PhD and colleagues at the Seattle Children’s Research Institute and the University of Washington [23-25]. The standard FMF Program integrates clinical techniques of positive behavior support, cognitive behavioral techniques, and motivational interviewing to improve primary outcomes of parenting efficacy, cognitive appraisal of the child and parent–child relationship, improving relevant knowledge, meeting family needs, and child behavior. On the basis of this theoretical framework, the FMF content, principles, and methods were successfully adapted to the mHealth format, with the addition of unique content and features [26].

In the second phase, most standard FMF Program content was preserved, but the flow of content delivery was adapted to be more amenable to self-direction by caregivers. There were additional adaptations from a technological perspective. In the third phase, FMF Connect was implemented by leveraging functionalities offered by modern mobile devices and by now ubiquitous access to the internet and Cloud services, as well as considering evolving technological possibilities and the different ways in which users interact with them. In the fourth phase, the initial interface design and functionalities were further refined through stakeholder input with caregivers in focus groups across 5 US cities [26].

This study focuses on the fifth phase of our user-centered design approach for systematic development and evaluation. This phase involves beta-testing of initial app prototypes followed by qualitative analysis of key informant interviews and data drawn from usage analytics to assess the feasibility of the intervention and guide further app refinements.

In this systematic model, the sixth phase involves pilot testing the intervention and trial procedures to establish the best methods for understanding the app in terms of the intervention process and outcomes. This stage is critical for optimizing the intervention and study methods for a larger-scale randomized controlled trial (RCT). The seventh phase involves a rigorous evaluation of app outcomes (and intervention process) through an RCT. Future studies will discuss findings from these phases of programmatic FMF Connect research.

### FMF Connect: A Novel mHealth Intervention

**Figure 2:**
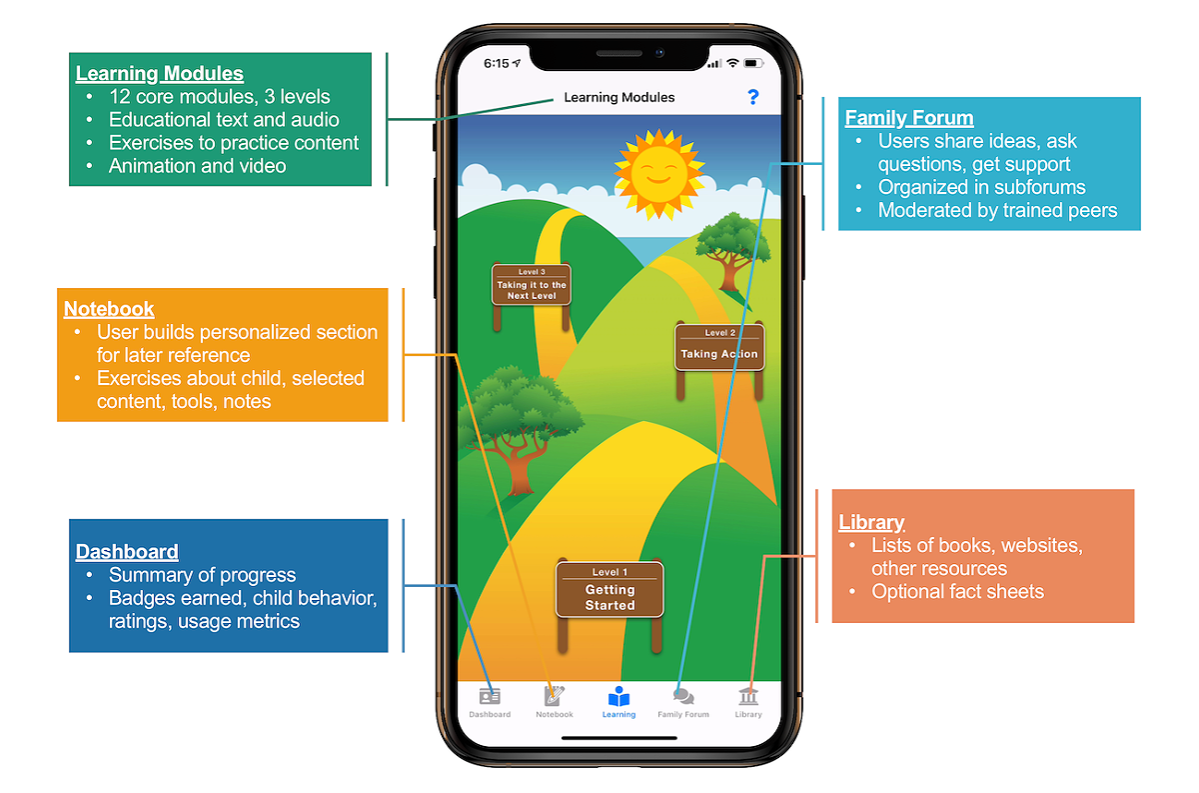
The 5 primary components of the Families Moving Forward Connect mobile health intervention for caregivers of children with fetal alcohol spectrum disorders.

The FMF Connect app consists of 5 primary components (Figure 2), which have been previously described in depth [26]. Briefly, the Learning Modules make up the core intervention and comprise 12 modules across three levels of educational content and skill development important in parenting children with FASD and behavioral concerns. In addition to brief educational text, the Learning Modules include exercises for active learning, and videos of real families demonstrating ideas and sharing their experiences. The Library contains additional videos augmenting those in the Learning Modules and fact sheets providing psychoeducation on important additional topics, such as mental health diagnoses, medication, trauma, advocacy, and resources. The Family Forum is a peer-moderated discussion forum where users can connect with others, share joys and challenges, and seek support or advice. Finally, the Notebook allows users to save content and exercises they wish to revisit later, and the Dashboard shows the user’s progress.

### This Study

In our systematic approach, the first four phases resulted in the development of FMF Connect app prototypes. In this study we report findings from the critical fifth phase, in which we conducted two rounds of beta-testing of the FMF Connect app prototypes to test feasibility; specifically, the feasibility and acceptability of the app intervention [27]. Each round of beta-testing involved 6-week trials, followed by key informant interviews and examination of app usage analytics. We engaged key stakeholders, including caregivers of children with FASD and medical, mental health, and other providers, to elicit their experiences and perspectives on the FMF Connect intervention. The feasibility trial was guided by the following objectives, informed by the Eldridge et al [27] conceptual framework:

1. Examine app usage data and crash reports to identify the required technological and functional refinements of the FMF Connect app.
2. Conduct focus group and individual interviews with participants to evaluate the user experience of the FMF Connect app to guide further app refinements.

The study results highlight important directions for the ongoing refinement of the FMF Connect app. By operationalizing a systematic model of app development and evaluation in this project, the findings also have broader implications for mHealth applications. Overall, this project offers generalizable ideas about methods for enhancing the acceptability and rigor of mHealth applications, a vital consideration as the field of digital health rapidly expands.

## Methods

### Study Design

This study was designed to assess the feasibility of initial prototypes of the FMF Connect intervention from both technological and user experience perspectives to guide further development of the app. This study involved two rounds of beta-testing, which allowed the examination of iterative feedback. The first round of beta-testing (BT1) was conducted from April to May 2019 and included the iOS prototype. The second round (BT2) was conducted from November to December 2019 and an updated iOS prototype and a new Android prototype with the same content and features were tested. Each beta test lasted approximately 6 weeks and included caregivers of children with FASD and providers working with this population.

To address the study objectives, this study used a concurrent quasi-mixed-methods design with equal priority given to both method types [28]. In other words, both quantitative and qualitative analytical methods were used. However, these methods were used to answer different aspects of the research question (ie, the feasibility of FMF Connect intervention prototypes). Therefore, deliberate integration of findings during the interpretation of results was not warranted [29]. After each 6-week trial, focus group and individual interviews were used to elicit participants’ perspectives about the app (qualitative data). Usage data and crash reports were also collected within the app and used to assess the functionality of the app (quantitative data). All study procedures were approved by the university’s institutional review board before initiation.

### Participants

A total of 63 participants (45/63, 71% caregivers; 18/63, 29% providers) were enrolled as described below by participant type. Table 1 describes the demographic information. Participants resided in 18 US states.

**Table 1:** Participant characteristics (N=63).

Characteristics				Caregiver (n=45)		Provider (n=18)
**Gender, n (%)**						
	Female		42 (93.3)		17 (94.4)	
**Age (years)**						
	Mean (SD)		50.41 (11.33)		45.28 (11.60)	
	Range		31-73		28-70	
**Average age of children with FASD^a^ (n=64)**						
	Mean (SD)		9.70 (3.58)		N/A^b^	
	Range		4-17		N/A	
**Caregiver ethnicity, n (%)**						
	Hispanic or Latinx		3 (6.7)		2 (11.1)	
**Caregiver race, n (%)**						
	African American or Black		4 (8.9)		0 (0.0)	
	Asian		0 (0.0)		1 (5.6)	
	White		40 (88.9)		17 (94.4)	
	Native American or Alaska native		2 (4.4)		0 (0.0)	
	Native Hawaiian or Pacific islander		0 (0.0)		1 (5.6)	
**Education level, n (%)**						
	High-school diploma or GED^c^		2 (4.4)		0 (0.0)	
	Some college, trade school, or Associate’s degree		13 (28.9)		0 (0.0)	
	Bachelor’s degree		12 (26.7)		2 (11.1)	
	Master’s degree or higher		13 (28.9)		6 (33.3)	
	Doctoral or professional degree		5 (11.1)		10 (55.6)	
**Relation to child,^d^ n (%)**						
	Biological parent		1 (2.2)		N/A	
	Adoptive parent		32 (71.1)		N/A	
	Foster parent		1 (2.2)		N/A	
	Grandparent		9 (20.0)		N/A	
	Other relative		2 (4.4)		N/A	
**Family income (US $), n (%)**						
	15,000-24,999		2 (4.4)		N/A	
	25,000-34,999		3 (6.7)		N/A	
	35,000-49,999		4 (8.9)		N/A	
	50,00074,999		7 (15.6)		N/A	
	75,000-99,999		4 (8.9)		N/A	
	100,000-149,999		12 (26.7)		N/A	
	150,000 or more		9 (20.0)		N/A	
	Prefer not to answer		4 (8.9)		N/A	
**Community type, n (%)**						
	Rural		7 (15.6)		N/A	
	Suburban		33 (73.3)		N/A	
	Urban		5 (11.1)		N/A	
**Experience with standard FMF^e^ Program, n (%)^f^**				7 (15.6)		9 (50.0)
**Round of beta-testing, n (%)**						
		Beta test 1		21 (46.7)		17 (94.4)
		Beta test 2		24 (53.3)		1 (5.6)
**Operating system, n (%)**						
	iOS		36 (80.0)		17 (94.4)	
	Android		8 (17.8)		1 (5.6)	
**Comfort with technology^g^**						
	Mean (SD)		5.73 (1.32)		5.83 (0.92)	
	Range		1-7		4-7	

^a^Some caregivers had more than one child with fetal alcohol spectrum disorders.

^b^N/A: not applicable.

^c^GED: General Educational Development.

^d^A total of three biological parents participated in the study. Two participants were biological parents but were noted in other categories (eg, grandparent, who was also a biological parent).

^e^FMF: Families Moving Forward.

^f^For caregivers, experience with FMF denotes completing the FMF Program, and for providers denotes training and delivering the FMF Program.

^g^Comfort with technology was measured using a Likert scale ranging from 1 (low) to 7 (high).

#### Caregivers

Caregivers, including biological, foster, adoptive, and relative caregivers (Table 1), were recruited through multiple mechanisms. Information about the study was shared with providers affiliated with the Collaborative Initiative on FASD and with moderators of national and regional FASD listservs and support groups, to be widely distributed to interested families. We also contacted eligible families in our university’s FASD research registry. Caregivers reported learning about the study from the following sources: provider referral (n=16), FASD research registry (n=3), national and regional FASD listservs (n=9), online support groups (n=5), and nonspecified (n=12). Caregivers were eligible for this study if they had a child with FASD or PAE between the age of 3 and 17 years and lived in the United States. Although FMF Connect is designed for caregivers of children aged 3-12 years, caregivers of adolescents (aged 13-17 years) were also included (n=9). These caregivers were able to reflect on their experiences in parenting their children across the full age range targeted by the app and evaluate the app in this context. A subsample of caregivers (n=7) who had previously completed the standard FMF Program was specifically recruited for this study. These caregivers could offer important insights on what it is like to learn this content in a self-directed manner through FMF Connect versus their prior lived experience of participating in the in-person, therapist-led standard FMF Program.

#### Providers

Although providers serving children with FASD and their families (eg, medical and mental health providers, occupational therapists, and advocates or educators) are not intended to be direct consumers of the FMF Connect intervention, there were several important reasons to solicit their feedback. First, many serve a diverse range of families and could offer insights to augment those provided by caregivers enrolled in the study. In addition, providers are likely to be a primary future referral source for the FMF Connect app. Gaining their perspective early in development may facilitate app acceptability so that providers will more likely share it with families once it is widely available. In this study, providers working with children with FASD were purposefully sampled through known provider networks relevant to this population. Providers were eligible for this study if they served children with FASD and their families and worked in one of these professions: medical provider (5/18, 28%), mental health providers (8/18, 44%), occupational therapists (2/18, 11%), and FASD advocates or special educators (3/18, 17%). A subsample of providers with experience delivering the standard FMF Program (9/18, 50%) was specifically targeted for this study.

### Procedures

Interested participants were sent the study consent form and demographic questionnaire. Informed consent was then completed with the study coordinator over Zoom (Health Insurance Portability and Accountability Act–compliant) or via phone. Participants returned the signed consent and demographic form before receiving the app prototype and installation instructions.

During the 6-week beta tests, participants could use the app at their discretion. As part of the intervention, the participants received weekly emails. These highlighted specific app features and content and provided information on technical assistance access. The Family Forum was moderated by 2 experienced caregivers who had previously completed the standard FMF Program and were supervised weekly by the first author. The study team monitored use and metrics throughout each trial. Bugs and crashes were tracked, and updates were sent to address problems or expand functionalities.

Following each 6-week trial, participants were asked to complete individual or focus group interviews with a member of the study team. In BT1, focus groups were organized by participant type and usage pattern (eg, number of modules completed, relative time spent in the app). Interviews were offered to participants when focus group participation was not possible because of schedule conflicts or comfort levels. For logistical reasons, planned individual interviews were conducted with all BT2 participants. Data collection was completed via Zoom for all but one BT2 caregiver participant, who preferred an in-person interview to better accommodate hearing loss. The questioning route (details provided in Multimedia Appendix 1, Table S1) was similar across both beta tests. However, two topics were added to BT2 to assess participants’ perspectives on new features. Topics included Global Impressions & Experiences; Usage/Engagement; Technology; Utility; and Experience with Individual Components (eg, Learning Modules and Family Forum). After introducing the Global Impressions & Experiences topic, interviewers were given flexibility regarding the order in which they covered subsequent topics. This was done to facilitate conversational flow and follow the participant’s lead during the discussion.

### Data Analysis

Audio and video recordings of individual and focus group interviews were transcribed by the research staff. Observations of nonverbals (eg, tone, affect, referencing app on phone) were integrated within each transcript. All transcripts were checked for accuracy and completeness. The data were then imported into Atlas.ti for coding and analysis. Four research team members conducted primary analyses: one of the principal investigators, a graduate student, and 2 research staff. All members of the analysis team were involved in data collection.

A thematic analysis [30,31] was undertaken to understand participants’ experiences using the app from both technological and content standpoints. Coding methods were selected a priori to inform further app refinements for subsequent larger-scale trials. These include structural, evaluation, and value coding [31,32]. Structural coding was used to delineate when participants discussed different app components (eg, Learning Modules and Family Forum). Evaluation coding was selected to identify participants’ positive or negative judgments about the FMF Connect app and recommendations for further improvements. Value coding was used to identify caregiver values, attitudes, and beliefs related to the experiences of raising a child with FASD and using the FMF Connect app. For provider data, value coding was only used when providers spoke about their perceptions of the values, attitudes, and beliefs of caregivers.

Systematic thematic coding of transcripts was completed between May and December 2020. Four parent interviews from BT1 were randomly selected and independently coded line by line by all 4 coders to establish the study codebook. Weekly meetings were held to establish consensus and operationalize first-level codes. The remaining BT1 caregiver interviews were then distributed across coders, taking care not to assign transcripts to the team members who had conducted the interviews. Weekly coding meetings were held to address any coder questions or suggestions for new codes.

Following completion of BT1 parent interviews, the team engaged in code mapping to organize and consolidate first-level codes into preliminary second-level pattern codes to facilitate subsequent coding [31,32]. BT1 provider coding and BT2 coding followed the same process. The preliminary second-level pattern codes represented the data well across BT1 providers and all participants in BT2. No new second-level pattern codes were added across these participants, suggesting adequate data saturation and consistency across trials.

Participant matrices [31] were used to examine variance in second-level pattern codes across participants and several key demographic features (eg, prior participation in FMF, BT1 vs BT2). Participant demographic variables were imported into Atlas.ti, and code co-occurrence tables were examined to assist with this process. Team members iteratively consolidated and interpreted the connections among the data through analytic memo writing to derive the final analytic model.

App usage metrics were examined for caregivers. Usage data were extracted from the cloud services used for the app. Descriptive statistics were calculated for several indices (eg, number of modules completed, number of posts in the Family Forum, and total time spent in the app). Learning Module completion patterns were examined using graphical methods.

## Results

### Objective 1: Examine App Usage Data Metrics to Identify Any Needed Functional Refinements to FMF Connect

#### Overview

Table 1 provides the breakdown of participants and the type of operating system across the beta tests. A total of 84% (38/45) of parents (BT1=16/20; BT2=22/25) and 94% (17/18) of providers (94%; BT1=16/17; BT2 1/1) installed the FMF Connect app. In BT1, 4 updates of the iOS app were distributed; in addition to bug fixes and performance improvements, updates included the ability to see if there were new posts in the Family Forum since the last user’s login, the addition of the Profile Graph Tool, and improvements in the screen unlocking experience and avatar customization. In BT2, 3 updates of the iOS app and 2 updates of the Android app were distributed for bug fixes and performance improvements.

On use days, caregivers averaged approximately 20 minutes (mean 19.63, SD 19.59 minutes) using the app. The largest amount of time was spent watching videos in the Learning Modules (45% on average). In the Family Forum, there were 54 original posts in BT1 and 45 posts in BT2. A total of 67% (30/45) of users posted at least once in the forum.

Not unexpectedly, patterns of use varied considerably among caregivers. The standard FMF Program is similar in total time spent on other parent training programs. The FMF Program typically involves 6 to 9 months of therapist-delivered content in a collaborative therapeutic relationship with caregivers (sessions every other week). Therefore, we did not necessarily expect users to complete all 12 modules in the initial 6-week test. Usage data differed according to the operating system and will be discussed separately.

#### iOS Usage

**Figure 3:**
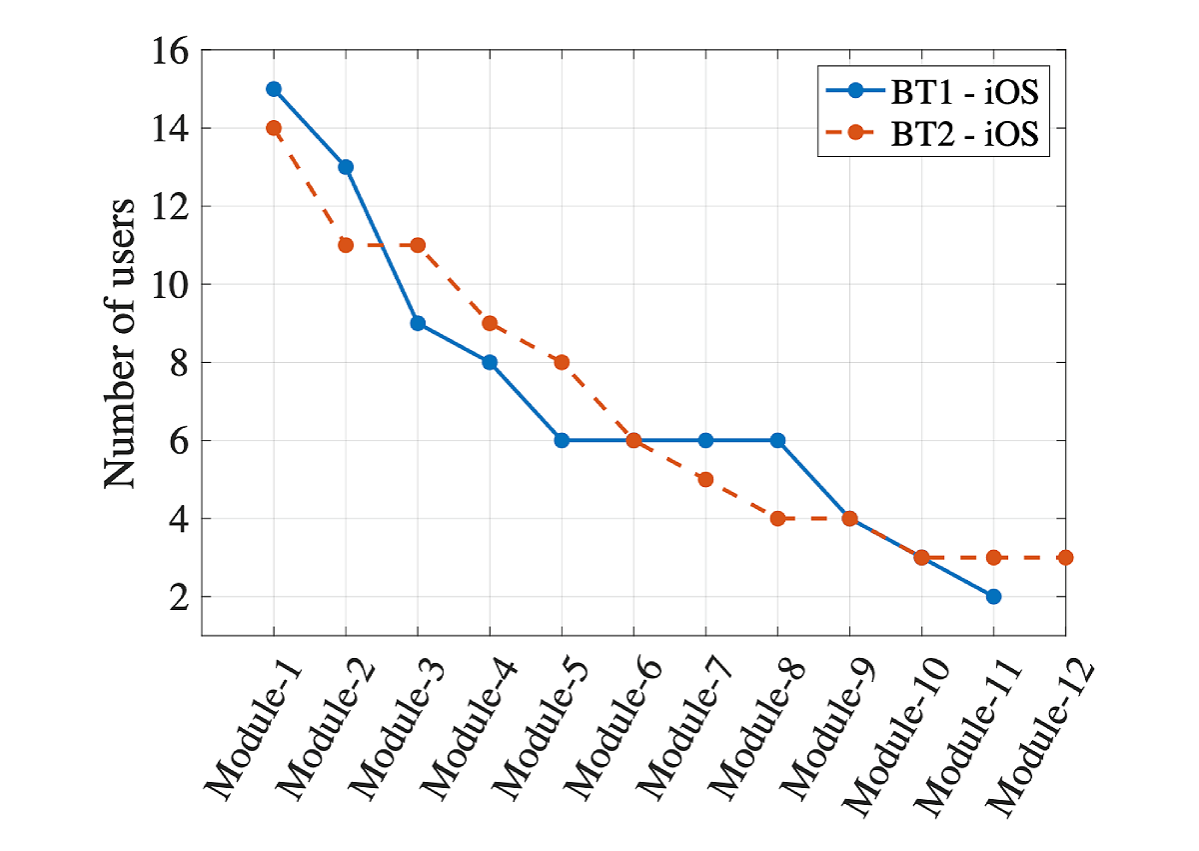
The number of Learning Modules completed by caregivers using Families Moving Forward Connect on iOS phones by beta test. The first round of beta-testing (BT1) had 4 caregivers who did not install the app and 1 who installed but had no module completion. The second round of beta-testing (BT2) had 2 caregivers who did not install the app and 2 who installed but had no module completion.

**Figure 4:**
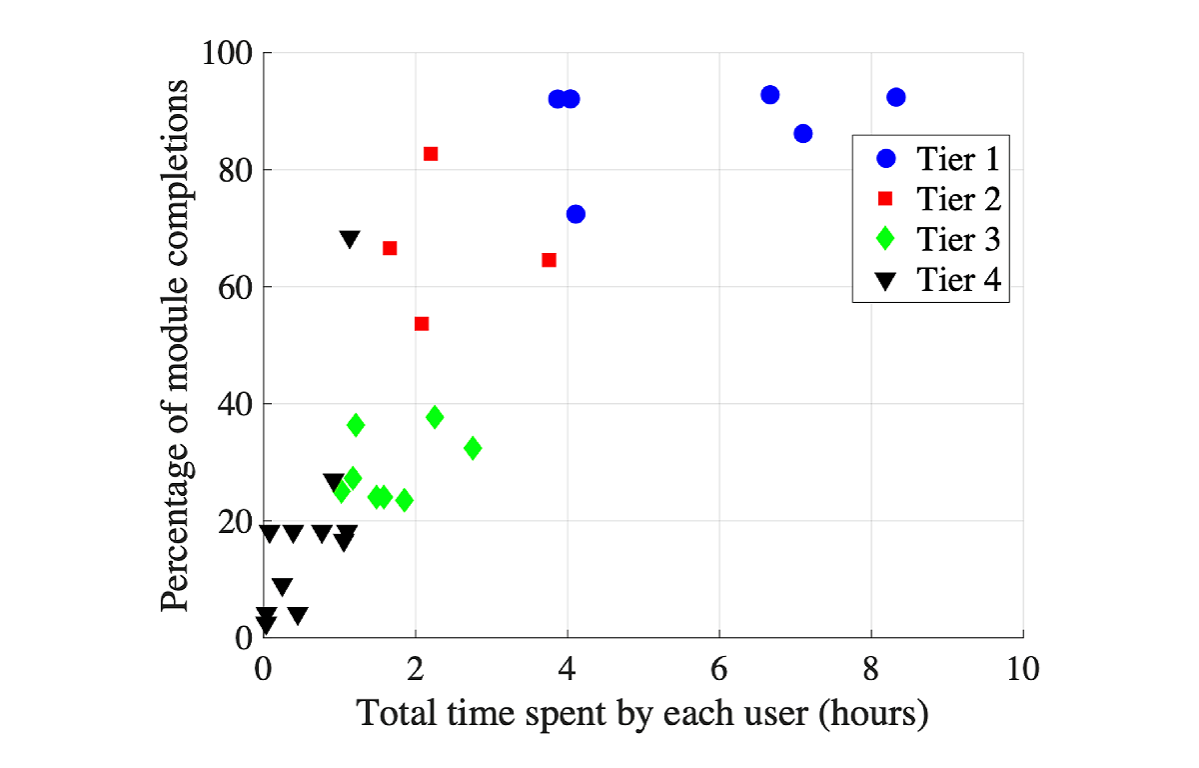
Tier classifications of usage for iOS users of the Families Moving Forward Connect mobile health intervention.

**Figure 5:**
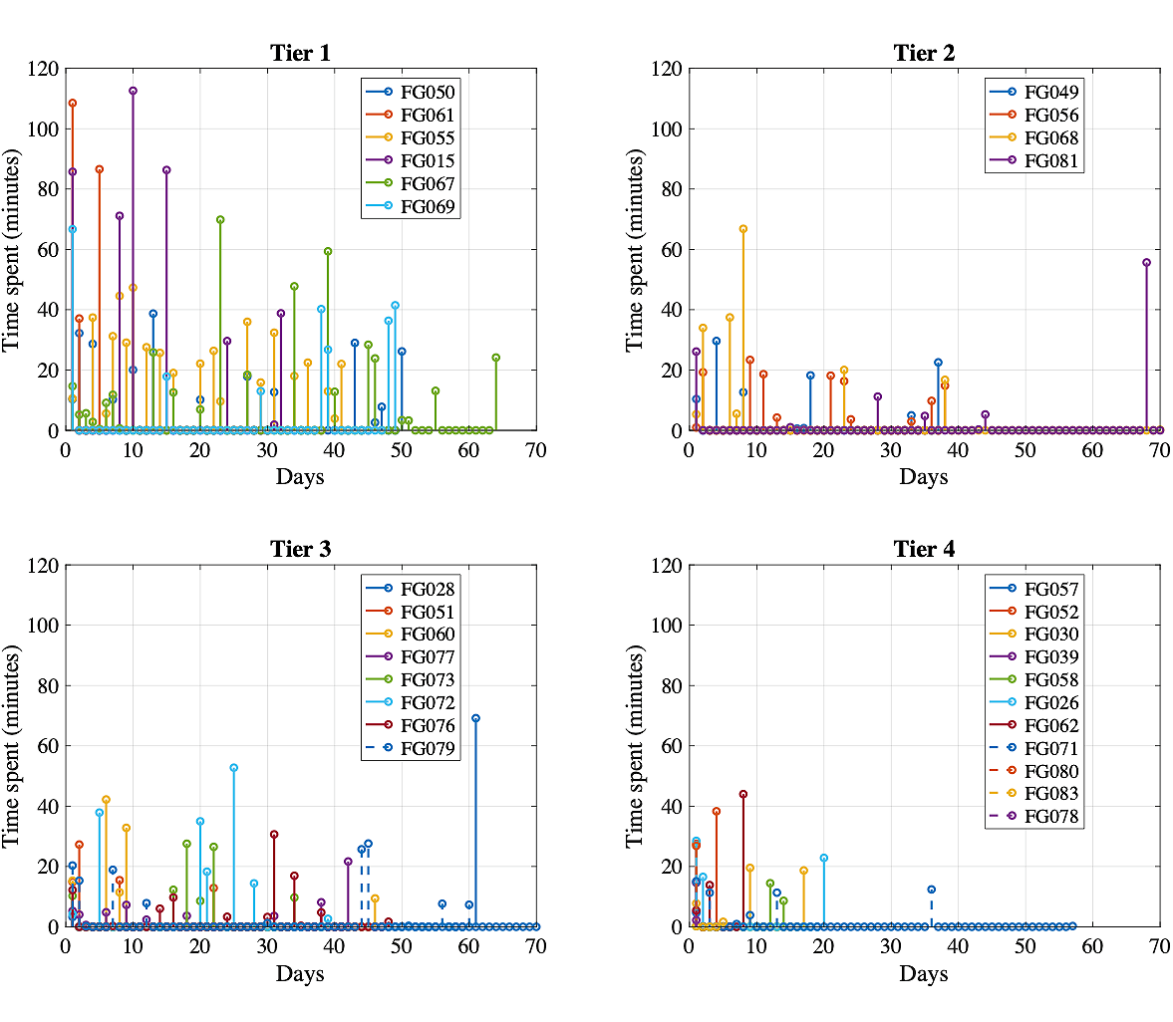
Total time spent in Learning Modules by day of use for iOS users grouped by usage tier.

Figure 3 shows the number of Learning Modules completed by iOS users by beta test. A total of 31% (10/32) of iOS users who installed the app completed at least through module 6 (an average of 1 module per week). We also examined the time spent on each activity within the modules. Bar graphs for each module were created with minutes spent in sections by the user (not shown). Through visual inspection, 5 usage tiers were characterized based on time devoted to activity completion and conceptual organization of modules, ranging from tier 1=higher robust use to tier 5=installed but no module use (Table 2 provides descriptions and number of users per tier). Figure 4 shows this classification graphically. Graphs were also created for Learning Module total time per day to illustrate usage patterns by tier (Figure 5).

**Table 2:** Learning module usage tiers for iOS users who installed the Families Moving Forward Connect app.^a^

Tier	Description	iOS users (n=32), n (%)
1	Higher robust use: Completed at least up through module 9 (level 3) or finished all modules Generally adequate time to complete activities	6 (19)
2	Moderate use: Completed at least up through module 6 (level 2) with adequate time to complete core activities May have had some variable usage (eg, skipping through activities) in some sections or modules after 6	4 (13)
3	Good level 1 use, but drop off: Demonstrated adequate time to complete activities in modules 1-3 or 1-4 (level 1)	8 (25)
4	Minimal or low use: Only completed up through modules 1 or 2, or Inadequate time to review information or complete activities (skipped through screens)	11 (34)
5	Installed but no module use	3 (9)

^a^6 iOS users did not install the app; 4 were in first round of beta-testing, and 2 were in the second round of beta-testing.

Tier 1 users tended to distribute their use of the app across regular sessions of 15-30 minutes or several very lengthy sessions of 60-120 minutes toward the beginning of the trial; total app use time was between 4 and 8 hours for these users. Tiers 2 and 3 users tended to use the app more sporadically and for a shorter period than those in tier 1. A few users also had a spike of high use toward the end of the trial, which likely coincided with scheduling a study interview. Many tier 4 users logged in just once or twice. The 2 tier 4 users with a higher percentage module completion quickly skipped through most screens.

iOS usage tier membership was also examined by participant characteristics through visual inspection and statistics (chi-square and analysis of variance). Usage tier did not differ by caregiver age (*F*_3_=1.41; *P*=.26), level of education (χ^2^_9_=6.15; *P*=.73), previous receipt of standard FMF (χ^2^_3_=2.77, *P*=.43), target child age (*F*_3_=2.46; *P*=.09), or income level (χ^2^_18_=23.17, *P*=.18). Tiers varied according to comfort with technology (*F*_3_=9.07*, P*<.001). Participants who rated themselves lower on comfort with technology were surprisingly more likely to be in tier 1.

#### Android Usage

Given the small size of the development team, the Android prototype was implemented on a schedule deliberately set behind the iOS prototype, and not all functionalities were available at the time of beta-testing. Nevertheless, the app was distributed to Android users for the initial testing of the technology. Usage patterns for Android users in BT2 differed from those of iOS users across tests, likely because of technical problems with the Android prototype. Incomplete data appear to have been recorded in cloud services for Android users. This may have occurred because of synchronization issues after users completed modules offline. The data recorded show that 3 of the 6 users completed at least some (but likely all) of each of the first 4 modules. The other 3 users only have data recorded for some of module 3. Toward the end of BT2, a few users reported that they could not unlock modules in level 2 of the app. It is possible that these users would have proceeded further in using the app if they had not encountered this technical barrier.

### Objective 2: Using Qualitative Methods, Evaluate the User Experience of FMF Connect to Guide Further App Refinements

#### Overview

We attempted to interview all study participants regardless of whether they installed the app or their usage pattern. Focus group and interview data were available for 26 parents (BT1: 3 in focus groups, 7 interviews; BT2: 16 interviews) and 16 providers (BT1: 6 in focus groups; 9 interviews; BT2: 1 interview). Of the 19 caregivers not interviewed, nearly all had little to no app use (1 in tier 2; 8 in tier 4; 3 installed but no use; 7 did not install). Themes did not generally differ between iOS and Android users, with the exception of ease of use and technological problems noted below.

General comments on the app were the most frequent (166 coded segments). Following this, the Learning Modules (151 coded segments) and Videos (106 coded segments) garnered the most discussion across participants, with often lengthy, detail-rich segments. The Family Forum (94 coded segments) and Library (59 coded segments) received a modest amount of discussion, with the Notebook (32 coded segments), Dashboard (32 coded segments), and Logo/Icon (11 coded segments) eliciting limited discussion of short duration.

#### Findings From Values Coding

Although not explicitly queried in the questioning route, caregivers often spoke of their attitudes, beliefs, and values. These provide an important context for understanding their evaluation of the FMF Connect intervention. Themes did not vary by round of beta-testing, Learning Module usage tier, smartphone operating system, caregiver type (adoptive vs relative vs biological), child age (child vs teen), or previous standard FMF Program experience.

FASD are often described as complex and confusing. For example, one caregiver (FG028) shared the following:

I remember the early days and thinking, “What on God’s green Earth, you know, is wrong with this child? What is going on?”... umm, “You could do this yesterday, what do you mean you don’t know where your shoes are? (Voice raises) How do you put your shoes on the wrong feet 90 percent of the time? How does that happen (laughs)?”...the behavior is just baffling in the beginning.

The complexity of FASD is further complicated by the fact that many children with FASD have experienced trauma and have other comorbid conditions, as illustrated by a caregiver of teenagers (FG079):

That was our struggle with our kids when they were little. Is it because of their alcohol exposure? Is it because of the trauma? Is it because of who they are? Is it a mental health thing? And, you know, everyone has their own opinion when you take them to therapists and doctors.

Participants emphasized that FASD information and resources are often lacking, which is associated with feelings of frustration, grief, and being overwhelmed. For example, one provider (FG059) stated as follows:

So many parents are desperate for answers, they’re desperate for information. ... There’s a lack of resources and lack of evidence-based intervention in most communities.

A caregiver (FG043) also emphasized difficult emotions arising from inadequate supports:

There is great remorse and guilt... I had a child with FASD because no physician took the time to get me into treatment when it was very obvious that I needed treatment.

Parents further described FASD as isolating. For example, a caregiver (FG049) described the following:

I don't have the opportunity to talk to other parents...umm...ever (laughs) who have children with FASDs, umm, so that is very isolating. … Because we (raises volume) can't even find a doctor who knows what they're talking about, let (normal volume) alone, umm, another grown-up going through it.

Caregivers spoke to their desire to do anything to help their children be as successful and independent as possible. Given the limited number of knowledgeable and skilled professionals, this often results in the need to educate others about FASD. For example, a caregiver (FG065) explained as follows:

We, as parents, you’re always educating other people. And so teachers and parents,...some doctors...anybody working with your kid, you know, occupational therapist, speech therapist, therapist...(emphasis) A lot of people do not understand.

As a result, caregivers raising children with FASD highly value access to information about FASD, people who understand their experiences, and the ability to connect and share resources with others. Participants expressed the belief that mHealth interventions, such as FMF Connect, are needed to help address barriers. For example, a caregiver (FG071) stated as follows:

We don’t have anything to really go to, so I think it’s really great to have the educational piece but also to have the forum, to kind of link people together because you do feel really isolated.

Many related these barriers to strong emotions, such as frustration. One caregiver (FG084) described the following:

A lot of people go on here to learn things. But, to be honest, (strong emphasis) I think most people go on the apps and go on the groups just to be with other people who are going through it…nobody in my life understands (frustrated tone)... And if I post in there, it would be mostly just to exhale to other people who get it, you know? And for somebody else to come on there and say, “I get it.” You know? (slow, normal volume) It’s just sometimes, that's all you need for the day. Is for somebody to say, “I get it.”

Because caregivers raising children with FASD are often overwhelmed, participants articulated the need to use their limited time wisely and valued choice and autonomy in self-directed learning. A provider (FG040) stated as follows:

You know, people are busy and they just-they want to know how much time these are going to take...knowing how much time you generally might need to spend with something… just might keep people engaged.

A caregiver (FG051) emphasized the value of choice:

I think the more people can make choices in what they’re doing, the more the buy in and (laughs) you know, the more likely they’re gonna do it and, and be happy about doing it.

#### Evaluative Coding: Positives

Across participants, there were 2.5 times as many positively evaluated coded segments as negatively evaluated segments. The vast majority of themes were consistent across demographic and usage tier variables. The few differences are discussed in the relevant sections.

##### Global Impressions

Global impressions of the app (eg, “I love it!” “It’s wonderful,” and “I enjoyed it.”) were nearly all positive (93 global positive vs 1 global negative segment). Participants appreciated the accessibility of the app and how they could fit it within their everyday lives. For example, a caregiver (FG015) said:

This makes it easy for me, it’s right there at my fingertips.

A provider (FG032) also highlighted the benefits of FMF Connect as a smartphone app:

The majority of my families do not have a home computer this year. The majority of them do everything off of their cell phone so that’s their only access to the internet.

Most of the iOS users across both beta tests also commented that they found the app easy to use. In contrast, most Android users did not mention this theme, likely because of technological problems in the Android prototype. The participants also made positive comments about the app’s appearance.

##### Learning Modules and Libraries

Positive evaluations of the informational content provided by the app had the most coded segments across all codes (139 segments). In fact, every parent made at least one positive comment regarding the informational content of the app. Participants appreciated how the FMF Connect app made this information more accessible to them. For example, a caregiver (FG056) explained:

It’s hard to find good information on FASD, and I thought that it was kind of cool that it was on my phone, all together, in one spot.

Several participants spoke about the quality of information. For example, a caregiver (FG065) stated:

I thought it was all very relevant and research based which I appreciate (laughs) very much.

Informational content also overlapped with parents’ values of understanding their children. Several parents provided specific examples of how app content helped them better understand their children’s behavior and respond differently. Participants were especially enthusiastic about the ability to share information from the app, particularly with teachers (relating to the themes of needing to educate others and value of sharing resources). For example, a caregiver (FG079) described as follows:

I printed out something to take to her meeting that I have next week for the teachers. … I think that was that was the big thing that I was excited to find this stuff to give to them.

Participants also spoke positively about aspects of the videos, including diverse representation of families, range of child ages and degree of problems, and specific ideas and strategies to try. Caregivers especially appreciated that the videos featured real families, as illustrated by a caregiver (FG056):

I was like, really excited when I first started and I was watching the videos and I was like “Oh my gosh!” You don’t get to see how other FASD kids live and how they are, so it was really cool to see like real families and real kids. Like, that was my personal favorite part of the whole thing.

Positive comments about the videos also often co-occurred with themes of parents feeling less isolated and validated in their experiences as parents. For example, a caregiver (FG052) shared the following:

[The videos] kept me grounded and mindful, umm, that, first I’m not in this alone. And other people are experiencing the same thing, and here are some things that they found that worked.

The exercises within the Learning Modules received positive evaluations by some users. Some participants commented on how the various exercises and games also helped them reflect on learning content and apply information to their children.

Similar to feedback revealed in prior studies of FMF Connect [26], the step-by-step progression of access to content in the Learning Modules and Library received mixed evaluation. Discussion of this theme was often intertwined with participants’ previous knowledge and experience with FASD and thoughts about whom the app is best suited. Every provider, especially those trained in the standard FMF Program, spoke to the need for and positive aspects of the step-by-step progression of these components. Most caregivers also spoke of the advantages of step-by-step progression. A caregiver (FG050) stated:

I liked how the progression went. I thought it was easier to be able to focus and break it down and think about that particular section at a time.

Several parents emphasized how this progression made learning less overwhelming. Although less enthusiastic about the step-by-step progression for themselves, more experienced parents felt this would be very beneficial for parents of newly diagnosed children and thought the app was most well suited for this group. For example, a caregiver (FG028) stated as follows:

I’ve been in the trenches for many years (laughs). It’s nothing is new to me. And I know-like, I guess I kept thinking as I was using it, this would be super helpful for someone who was in my shoes 8 years ago.

Participants also spoke to the benefits of the FMF Connect app as a *refresher*. They described how parents could go back at any time and review content and apply it to new challenges. For example, one caregiver (FG061) stated as follows:

I have thoroughly enjoyed all of the little activities we had to do that reinforced everything… Being able to go back to see it over and over again. To get it going in your little brain “Ok, I can, I can do this.”

##### Family Forum

Participants were positive about the inclusion of the Family Forum and articulated its potential to reduce isolation and help parents connect with others who have shared experience. Evaluations of the Family Forum often overlapped with parents’ values of connection and people who understand. For example, a caregiver (FG025) explained as follows:

I thought it was useful that people could ask questions, like the real issues that we deal with. And get some kind of advice and some kind of help because I find that we deal with things that most people aren’t dealing with all the time.

Participants particularly liked that the Family Forum was moderated and that there was a special section where their posts were saved for later reference.

##### Dashboard and Notebook

The Dashboard and Notebook received fairly limited discussions and were primarily associated with nonspecific positive impressions. However, during beta-testing, these components had relatively limited functionality. In BT2, a Tip of the Day feature was added through a push notification that subsequently appeared on the Dashboard for that day. Enthusiasm was high for this feature, and nearly every participant in BT2 provided a positive evaluation. For example, a caregiver (FG042) explained as follows:

The constant tips - I mean it’s like having a social worker right in your home with you all the time … It doesn’t matter if you’re having a good day or a tough day, having that positive reframing and, it, it’s like a breath of fresh air, it’s like, ok, slow down, you know this, and here’s a reminder, yes, ok, (laughs) you have to let go of that and you have to do what the tip says.

Participants also described how the Tip of the Day was useful in reminding them to use the app.

##### Motivators and Facilitators of App Use

Participants identified app content and the ability to connect with others as primary motivators of wanting to use the app. One caregiver (FG072) described this as follows:

That's a big motivator, too, is just wanting to have one more tool. I have the books. I'm watching the YouTube videos. I'm doing everything that I can do. But I have my phone with me all the time.

Another caregiver (FG068) shared as follows:

I think for a lot of parents the motivation [to use the app] would be just, you know getting help…And connect with others.…Cause it’s hard when other people don’t understand.

Parents described using the app most often at night once their children were in bed or during moments of downtime. For example, one caregiver (FG050) used the app:

Whenever I had free time. Usually, before the kids woke up, or after they went to bed. So it was just, whenever I had time, I would do it for a couple minutes here, a couple minutes there.

The parents who progressed the furthest in the Learning Modules (ie, tier 1) described strategically planning ahead for manageable segments of time to work through the app. One caregiver (FG061) described this as follows:

I’d spend at least thirty minutes every day, if not an hour, if I had the time, I’d make sure I had the time, but not everybody has my schedule.

#### Evaluative Coding: Negatives

##### Overview

Negative evaluations comprised 28.36% (312/1100) of the total number of evaluation segments coded, so the study methods were successful in eliciting these. These negative evaluations largely emphasized technological issues, constructive feedback relative to navigating the app, and barriers to use. The only negative global impression segment across all participants was from an Android user (FG066) who experienced difficulties loading the videos and was disappointed by the level of activity in the forum:

Um, (laughter) to be honest I wasn’t very fond of [the app]... Um, granted there were very few people on as testers, but… I didn’t see a whole lot of conversations going on. Um, its, the videos themselves, half the time they didn’t work for me.

##### Technological Problems

Some users experienced technological problems using the app. These were the most significant for iOS tier 4 and Android users. BT1 included all iOS users. In BT1, some participants experienced confusion or difficulty with TestFlight (an iOS app that allows beta-testing before release in the App Store). For these users, difficulty with TestFlight impacted the initial installation or updating the app. Several updates were released early in BT1 because of some crashing and videos not loading consistently. In addition, several participants reported some difficulties in saving their progress in the Learning Modules; after refinements, this was not an issue reported in BT2.

Android users (who were only part of BT2) described more significant technological problems that resulted in barriers to using the app. These issues included app freezing, some inaccessible links, and problems loading videos or unlocking later content. For example, one caregiver (FG066) described this as follows:

In this app, it was just stuck, I couldn’t get out of it, and I couldn’t do anything unless I completely closed it down.

A provider (FG036) reported problems with video loading:

I could not get the first video to play. Umm, and that was something that had happened throughout, like me trying to watch the videos is they just keep buffering and buffering.

##### Navigation: Family Forum

**Figure 6:**
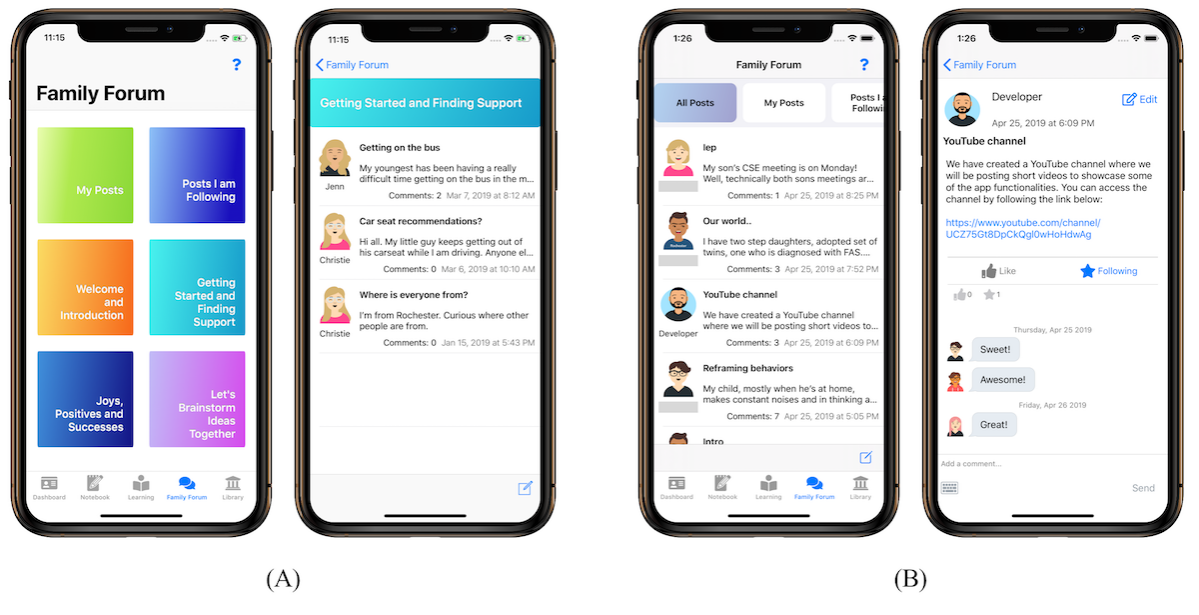
Families Moving Forward Connect interface design changes for the Family Forum during (A) round 1 of beta-testing (BT1) to (B) round 2 of beta-testing (BT2).

On the basis of previous feedback regarding the design of the FMF Connect app [26], the Family Forum was initially laid out to allow gradual access to subforums tied to Learning Module completion to support privacy and shared knowledge. However, participants in BT1 did not find the Family Forum interface to be very intuitive (Figure 6A). A caregiver (FG050) explained as follows:

I found that there’s too many boxes. There’s too many sections and to go check on each one and to see what people wrote and see what their comments are as opposed to Facebook’s way, like everything is there.

On the basis of this feedback, the Family Forum interface was redesigned for BT2. Subsequently, there were fewer negative evaluations related to navigating the Family Forum (Figure 6B). In BT2, negative evaluations mainly centered on parents not noticing or understanding the tags or categories at the top of the screen.

Participants also noted that the overall use of the Family Forum was lower than expected, and conversations tended to stagnate because of insufficient notifications. For example, a caregiver in BT1 (FG051) commented as follows:

I went in and I introduced myself, and I was excited about the forums and connecting with other people but then, I think having so many different forums and not really getting notifications of when people were posting, umm, that made me just keep forgetting about them.

##### Navigation: Learning Modules and Library

As mentioned previously, the step-by-step progression of the Learning Modules received a mixed evaluation. Reactions to move through the content in order varied by usage tier and previous experience with FASD. Some experienced users, with robust tier 1 use, identified that content was redundant for them. However, none of these experienced users described their experience as tedious or found that step-by-step progression was a barrier. These themes were only present for lower-use tiers 2 to 4. Negative evaluations related to step-by-step progression occurred more often for participants with a greater degree of previous knowledge and experience with FASD. One caregiver (FG066) expressed this sentiment as follows:

I’m just simply not going to sit through you know, 5, 10 hours whatever it is of information and watch it and everything when I already know it just to get to something I don’t know. There are better ways for me to do it.

Similarly, about the Library, a caregiver (FG069) explained as follows:

Maybe it’s because I already came in with a fair amount of knowledge, but I…wasn’t a big fan of different things opening up as I went. I would have preferred to have jumped in and seen everything immediately.

Participants in BT1 found the number of videos and screens per module to be overwhelming and a barrier to completion. For example, one caregiver (FG051) explained as follows:

I’d learn about something and then there would be people sharing their experiences, which is great. But after the first two or three I was like ok, I get it, and then there were like 12! (laughs)… It took more time than I maybe had at the moment, and I wanted to kind of get past the videos and work on whatever was next.

Given this feedback, refinements were made for BT2, which included a table of contents for each module with fewer screens per section. A smaller number of videos were customized for users on the basis of user-imputed data (eg, child age and behavior problems), and the remaining videos were stored in the Library for further viewing. These changes were very positively evaluated by the BT2 participants.

##### Barriers

Identified barriers to app use generally corresponded to the negative evaluations discussed above. Time and attentional resources were also described as barriers. One caregiver (FG062) explained as follows:

Basically I have free time for like 10-15 minutes at night when I’m putting the youngest kids to bed. And of that, I have very few minutes where I can actually listen.

Another caregiver (FG071) also commented as follows:

I think for me it was too much work at that time of the day… A big issue for me is knowing my energy level at that time of the day and knowing what I had to do.

Providers offered additional insights into potential barriers on the basis of their experiences working with families raising children with FASD. Several providers mentioned factors, such as age, literacy level, English as a second language, and comfort with technology as potential barriers for some families they work with. For example, a provider (FG047) stated:

I have other [patients] that are great-grandmas and grandmas who barely have a computer and have a cell phone mostly to accept phone calls, and make phone calls and “I don’t know about these apps honey I don’t want to deal with that.” And then of course a big barrier here …is we have a big Spanish speaking component.

Another potential barrier raised by providers was parents feeling intimidated or lacking confidence in implementing strategies demonstrated by parents in the videos. For example, a provider (FG032) described as follows:

As I’m watching the videos, I know some of the parents I’m working with would be petrified to just watch [child’s name]’s mom because they’re like, “I could never do that. I have six children. How is this going to work?”

#### Evaluative Coding: Recommendations

The participants offered a number of useful recommendations to improve navigation and enhance engagement with the app (Table 3). Some of these recommendations were directly related to aspects that were negatively evaluated. As mentioned above, these led to refinements to the app in between trials, and additional changes are underway.

**Table 3:** Recommendations offered by participants for further refinement of the Families Moving Forward Connect mobile health intervention.

Category		Recommendations
**General app-wide functions**		
	Engagement features	More robust notification system Coaching Changes to weekly emails Tip of the day
	Navigation—making things easier to find or use	Search function Overlays and tutorials Navigation shortcuts Multiple platforms (ie, phone, computer, tablet)
	Broader access	Spanish language, closed captioning Functions to allow consideration of multiple children with FASD^a^ within the app Ability to share the app and other materials with others Companion apps for teachers, children/teens, and others
**Learning Modules and Library**		
	Navigation/organization	Table of contents for each module, with fewer screens per section Open access to all content from the start Consolidate or offer selected number of videos Fewer clicks to start videos Live links to other web sites Having content available on (1) app screens so easier to read and (2) PDF for easy sharing
	Content	More real-life videos and practical strategies Research summaries Information specific to birth parents and ways to deal with guilt, grief, shame, and stigma Additional ideas for self-care Tips for advocacy and navigating systems Strategies for facilitating social interactions/friendships Video examples of clinicians working with parents
Family Forum		Different forum interface Open access to subforums Discussion starters Regional or state subforums; Provider directories Ability to direct message other users
New features under development		Behavior tracker Coaching Daily ratings

^a^FASD: fetal alcohol spectrum disorders.

Additional recommendations were closely related to the values expressed by the participants. For example, caregivers emphasized the importance of using their limited time wisely and having choice and autonomy over their self-directed learning. As a result, they recommended open access to all content in the app from the start and tools to make it easier to *refresh* their learning when they needed it. For example, a caregiver (FG057) stated as follows:

I understand the whole idea with the yellow brick road, I think that’s great, but I think for me, I would like things that I could just tap on that road, to kind go back and forth in some other groups and videos and stuff, and kinda jump around a little bit more...the app was just leading down a one road, which is great, but sometimes I like to take the detour.

Another caregiver (FG055) spoke to the benefit of repetition and refreshing her learning:

I think it would take a lot of repetition… for me to benefit the most from all the exercises. So, I think it would be nice … to do the same exercises over and over and over and over and over again. Especially when the behavior has just happened, and I want to go back and I want to do that exercise for that behavior.

Caregivers also value resources to help them better understand their children. Most caregivers liked the idea of a behavior tracker to help them monitor and notice patterns in complex behavior problems.

A number of recommendations related to a cluster of attitudes, beliefs, and values; specifically, caregivers’ need to understand their child, educate others about FASD, and connect with people who understand. For example, the recommendation for state or regional subforums in the Family Forum would help caregivers rely on others in their area to identify and share available resources and connect locally. One caregiver (FG049) stated as follows:

I have been in the hunt of my life trying to find my daughter just somebody to take her to that has even a basic understanding of her diagnosis. So, if I had like, like just a place where I could go and know like, “These are the people from my state.” Like, “Where do you take your child?”

Some participants also liked the idea of having a coach or expert associated with the app who could help them understand FASD, connect them with needed resources, and provide feedback and discussion relevant to their child. They recommended additional content and tools that would make it easier to share information from the app with teachers, providers, and other people working with their children. Some participants went further and recommended separate apps or components specifically for use with their children or with teachers. For example, one caregiver (FG056) said as follows:

I really liked the material. My daughter’s teacher would benefit from- it would be cool to like, have her access it also.

Another caregiver (FG069) also noted as follows:

You know what I’d really like to see is, is this program targeted at medical providers!

On the basis of their experience with other apps, caregivers highlighted some additional facilitators that might help them engage more with the app. The most frequent suggestion was a more robust notification system. One caregiver (FG015) described this as follows:

With so much...going on, with the stress level and all this stuff happening, sometimes you get easily, so overwhelmed you don’t get a chance. If it’s not at my fingertips or not right there, then it’s out of sight, out of mind, you know.

Similarly, a caregiver (FG042) stated as follows:

I find it helpful when I get a little prompt in my text or in my email, just to remind me that, you know, the app is here and you can, you can just click from that email or that text and jump into the app.

Participants thought it would be best if the user could customize the type and frequency of notifications to their preferences.

Several biological parents emphasized the importance of the representation and education of their experience in reducing stigma. For example, one caregiver (FG084) explained as follows:

You don’t see too many statistics that talk about successful parenting... by biological [parents], you know what I mean? ...And so, I tried to...I tried to be the best mom I can and I try to show people.

Another caregiver (FG043) described the following:

Birth parents understand why people are angry and they just want to prevent the next birth mother from drinking. But I think [education about why people continue to drink] would make them feel more welcome because we all understand that the general public doesn’t get alcohol use disorders. … Having that education, you know, education is the key.

One caregiver (FG084) emphasized the importance of the moderator in creating a welcoming space:

When I did my introduction, I was a little worried. Umm, the moderators were the only ones who welcomed me and that’s okay. I knew that was probably going to happen... I did post a couple of times… But nobody made me feel unwelcomed... and that is more important.

A caregiver (FG066) also made several specific recommendations for integrating additional support for biological parents:

Granted everybody’s in [the Forum] together. Maybe there would be an area that (pause) birth moms could go do, specific. That, not necessarily saying they’re any different from the other moms, because everybody’s a mom whether by choice or by birth, but more so, because birth moms often have the shame and the guilt associated that need to be worked through ...If you touched on it in the Learning Modules that would be great because a lot of women we find have a lot of guilt.

Providers also offered recommendations to increase accessibility for a broader range of diverse families, such as closed captioning and speech-to-text. Several providers also recommended Spanish-language features, given the large mono- and bilingual Spanish-speaking population they serve. Finally, both parents and providers mentioned that families often have multiple children with FASD and wanted features within the app to better accommodate this.

## Discussion

### Principal Findings

This study presents critical stakeholder feedback and usage data from two rounds of beta-testing of prototypes of the FMF Connect mHealth intervention for caregivers of children with FASD. This fifth phase in the systematic user-centered design approach to the development and evaluation process of the app (Figure 1) yielded important insights on the acceptability and usage patterns of FMF Connect. The findings have implications not only for subsequent app refinements specific to FMF Connect but also for broader mHealth and digital parenting and developmental disability-related interventions.

Two primary research objectives were examined to evaluate the feasibility of the FMF Connect intervention during this phase of our systematic approach.

#### Objective 1: Examine App Use Data Metrics to Identify Any Needed Functional Refinements to FMF Connect

First, we considered how well the app worked for diverse users from a technological standpoint. iOS users in both rounds of testing generally described the app as easy to use. Usage patterns were variable, but surprisingly, had few associations with demographic factors or how participants evaluated the app. Users in the “higher more robust use” tier 1 were more likely to strategically set aside time to engage with the app. Technical issues were more significant for users in lower usage iOS tiers. Recognizing technical issues during beta-testing, we released multiple updates in each trial to fix bugs and improve performance.

It should be noted that BT2 also included a new Android prototype that was designed in alignment with the iOS prototype. Despite alpha testing within the development group on several devices and simulators, Android users experienced greater technological difficulties. These included issues with loading the videos, unexpected crashes and issues when accessing some of the Learning Modules, and synchronization issues between the Android app and Cloud storage. Relative to iOS, it is possible that the more limited regulation and decreased consistency among Android devices and supported versions of the operating system contributed to these technological barriers. Consistent with the objective of beta-testing, we expected to identify technological issues in the context of real-world user implementation. This is a valuable part of the process of user-centered design and informs needed functional refinements.

Usage data also highlight the need to carefully consider design features for engagement and operationalize these features for the FMF Connect app. The tier classification of usage patterns in this study showed evidence of nonusage attrition, with 44% (14/32) of iOS users who installed the app with minimal or no use (tiers 4 or 5). Although this is within the range of premature dropout rates observed for in-person parenting interventions in community settings [33], much work is required to improve accessibility and engagement. Research has called for a *science of attrition* [34], arguing that understanding dropout and nonuse in mHealth interventions is essential to optimize interventions for targeted populations. In line with this, we examined participant characteristics across tiers and found that those with higher comfort with technology were more likely to have low or no use. It is possible that these participants had other supports in place or had already discovered the information on the web. This highlights the likelihood that the FMF Connect app is of particular importance for underserved populations and could help address social disparities.

More general data on usage patterns in mHealth and digital interventions highlight the critical need to carefully consider design features for engagement. On average, approximately 25% of apps are only used once after download [35], and only 29% of app users were still using an app 90 days after download [36]. Overall, these statistics suggest that engagement in self-directed mHealth and digital interventions can be challenging. This is especially true for parents, given the many demands on their time. Participants in this study noted barriers, such as lack of time and feeling exhausted and overwhelmed, which will arise for any parenting intervention. However, this may particularly be true for interventions targeting parents who are faced with the challenge of raising children with disabilities. A portion of the users in this study were able to strategically prioritize time to engage with the app on a regular basis. Clearly, additional features and supports are required to facilitate and maintain engagement for other users.

In a cogent review, Wei et al [37] identified seven themes that improve user engagement with mHealth applications. As shown in Table 4, the participants in this study independently identified and positively evaluated aspects of the FMF Connect app that correspond to each of the 7 engagement themes. Especially strong were features supporting the themes of interface esthetics, message presentation, and credibility. The findings reveal that many existing design features thought to enhance engagement were already built into the FMF Connect app. However, useful recommendations for further refinement relating to the four themes of navigation, personalization, reinforcement, and communication were suggested by participants.

**Table 4:** Comparison of design features of the Families Moving Forward (FMF) Connect mobile health intervention with thematic checklist of features to improve user engagement.

Design feature themes that improve engagement Wei et al [37]	Existing features in FMF Connect beta tests	Recommended features for future development
Interface esthetics	Pleasing color scheme Positive evaluation of graphics	—^a^
Navigation	Easy to use (iOS) Table of contents (BT2^b^)	Tutorials/overlays Direct search feature Navigation shortcuts
Personalization	Selected videos (BT2) Profile graph Exercises—provision of goal setting and feedback	Open access to content Multiple children Personalize notifications Behavior tracker tool
Reinforcement	Messages of congratulations Weekly emails Tip of the day (BT2)	Notifications Badges
Communication	Family Forum	Coaching State or regional forums
Message presentation	Simple language Positive and nonstigmatizing tone Videos Pictures Font sizes and colors to highlight information Checks for understanding in games/quizzes	Closed captioning Spanish language
Credibility	Evidence-based information from credible source Encrypted and password-protected	—

^a^None provided by participants.

^b^BT2: second round of beta-testing.

#### Objective 2: Using Qualitative Methods, Evaluate the User Experience of FMF Connect to Guide Further App Refinements

Next, we considered how users evaluated the FMF Connect intervention and what could be improved to enhance user experience before larger-scale testing. Despite differences in technological problems and usage metrics, iOS and Android users had remarkably similar evaluations of the app. Globally, users were very positive about the app, with 2.5 times more positive- than negative-coded segments across participants. Positive evaluations emphasized the need for and practical utility of the app, which often related to significant barriers to care in this population. The informational and video content of the app was described as particularly valuable to caregivers. The Learning Modules and videos yielded the most detailed discussion, and every caregiver made at least one positive evaluation of the content within the app, much of which was originally derived from the standard FMF Program.

Participants also spoke about the affective and social benefits of the app. They described that raising a child with FASD is a very confusing, frustrating, and isolating experience, which is consistent with previous literature [11,38,39]. Many positive evaluation themes were associated with caregivers’ expressed values of understanding their child and working to support their children’s success. Participants also valued watching videos of real families and connecting with others in the app, which made them feel validated and less isolated. Caregivers’ stated motivators for using the FMF Connect app consistently related to informational content and connecting with others, which aligns well with their values and beliefs.

Negative evaluations of the user experience largely emphasized the technical and navigational aspects of the app. On the basis of feedback from BT1 users, we redesigned the Family Forum interface and added organizational features (eg, table of contents) and tailored video presentation in the Learning Modules to improve navigation. These refinements were positively evaluated by most BT2 users. Consistent with our previous findings during the initial design process [26], the theme of step-by-step progression of learning content received mixed evaluations. All providers and most parents provided at least one positive evaluation of the step-by-step progression of content. Importantly, however, some users (especially more experienced caregivers in usage tiers 2-4) found this progression redundant or tedious. They preferred greater autonomy in self-directed learning, which characterizes the app. Step-by-step progression was identified as a barrier to the use of some caregivers. A recommendation for open access to content occurred frequently in relation to this theme. Time and attentional resources were common barriers to app use, as described by caregivers. Providers also offered insight into additional barriers that could impact caregiver use of the FMF Connect on the basis of their experiences serving diverse families, including literacy level, English as a second language, caregiver age, and comfort with technology.

Participants offered a large number of valuable recommendations for further app refinement to continue to improve the user experience. As described above, several of these were implemented in the period between BT1 and BT2 and were then favorably evaluated. Additional refinements, such as a behavior tracking tool, changes to weekly emails, and open access to content, have already been implemented and are being tested in a larger feasibility trial. Subsequent refinements, such as a more robust notification system, coaching infrastructure, search tools, overlays, integration of accessibility tools, and optimization of content for viewing and sharing are in progress.

### Relevance of Study Findings for Other Digital or mHealth Parenting Interventions

Only one other published study has systematically developed and elicited stakeholder feedback on a digital parenting intervention for FASD [40]. In an initial usability study of the Strongest Families intervention, which involved 11 web-based modules and weekly telephone calls, 8 caregivers and 10 providers provided feedback on the intervention across two cycles. Similar to FMF Connect, participants rated the Strongest Families website as appealing and relatively easy to use. Several usability issues were identified, including navigation, amount of content per page, and tailoring of content; these were subsequently refined with generally positive feedback. Together, both studies document the acceptability and feasibility of digital and mHealth interventions for caregivers raising children with FASD. RCTs are underway (Strongest Families; [41]) or planned later this year (FMF Connect). This systematic approach (Figure 1) may serve as a relevant model for the development of other digital and mHealth interventions.

This study demonstrates the benefits of considering the context of parenting values, beliefs, and attitudes when analyzing user evaluations of the FMF Connect app. Indeed, considering the relevant values, beliefs, and attitudes of caregivers will be informative when developing interventions for other clinical populations. Many values expressed by caregivers in this study have been reported by other parents of children with developmental disabilities [42-44]. Themes of needing to educate others and valuing people who understand are common in the developmental disabilities literature [42]. Current findings emphasize how much parents value access to information, especially because they report that many professionals cannot support them. Research suggests that access to information and services is very important for the well-being of parents of children with disabilities, with peers often being the preferred source of information [43,44]. One study found that parents of children with developmental disabilities felt judged and isolated, and often needed to educate others and seek out their own information. These experiences are major stressors for parents [45]. Although these parenting values are reflected in the broader disabilities literature, they may be especially the case in the field of FASD. Research has clearly shown that many professionals lack knowledge and training on FASD [7,46,47]. FASD can also carry stigma, which can lead to increased feelings of judgment and isolation [48,49].

With these points in mind, it is surprising that few digital interventions exist for parents of children with disabilities. Some preliminary evidence shows that website-delivered interventions are effective for parents of children with autism [50], but a significant need for evidence-based, accessible interventions remains. The accessibility of mHealth and digital interventions is responsive to some barriers to care and to the lived experience of parents raising children with disabilities [51]. Motivators of accessing information and connecting with other parents who understand, identified in this study, are likely to generalize to other populations, especially for those with low community awareness and limited access to care. The current findings demonstrate that choice and autonomy are also highly valued for self-directed learning, which is an important consideration for intervention design.

### Strengths and Limitations

This study represents the first initial test of a mHealth intervention for caregivers raising children with FASD, a part of a systematic approach to app development and evaluation. This study had many strengths, and efforts were made to reduce the impact of the limitations of this study. The findings emphasize the acceptability and feasibility of the FMF Connect app for caregivers and offer important directions for further refinement. This intervention has clear potential for larger-scale dissemination, with vital public health implications for this underserved population—and, perhaps, especially for some subgroups within this population facing greater social disparities. The methodological approach is also rigorous, involving iterative feedback from key stakeholders to ensure relevance and usability, which is an important step in user-centered design and development [20,52].

Study findings are limited by the perspectives of participants sampled. As is true in many studies, all participants were volunteers, contributing to the possibility of selection bias. The current sample size is relatively large for beta-testing feasibility studies and is considered sufficient for the primary objectives of this study. However, it is possible that valuable perspectives may have been missed. Although the consistency of themes was very high across both caregivers and providers, some demographics of this diverse clinical population are less well represented. For example, only 7% of the parents and 6% of the providers were men. Overrepresentation of women is common in studies involving primary caregivers [53,54]. The sample represented primarily adoptive parents, although the perspectives of relative caregivers and biological mothers are represented. Racial and ethnic diversity is also somewhat lower than in the general population, and all participants were English-speaking (although some may have had fluency in other languages). The study was also limited to participants who were able to afford smartphones, WiFi, or data plans. Participant income spanned all queried levels but was still skewed above average relative to national statistics. It is notable that the inclusion of provider perspectives provided additional insights into potential barriers and recommendations for families that may not have been represented in the sample.

Although our nonusage attrition rate was generally within the range seen in community and digital interventions, it may have contributed to bias in the study findings. Participants were less likely to complete focus group or individual interviews if they did not install the app or had low use. Therefore, the study of attrition factors was necessarily limited. Interviewees may have displayed a positive response bias. A number of participants expressed desperation for information and resources on FASD; therefore, fewer negative evaluations of the app may have been offered because of the lack of alternative treatments. The research team was also involved in developing the app, which could have impacted participant feedback. To reduce positive response bias, the research team actively tried to elicit negative and constructive feedback about the app and emphasized the benefits of hearing negative evaluations during this stage of development when refinements could more easily be made. Although significantly fewer than positive evaluations (n=788 segments), a robust number of negative evaluations were elicited (n=312 segments). However, it remains a possibility that users experienced difficulty expressing negative feedback in interviews, and this should be considered a limitation of the current work.

### Conclusions and Future Directions

This study demonstrates that a scalable digital health intervention can successfully be derived from an empirically supported therapist-led intervention tailored for families raising children with FASD, adding unique and important additional features. The FMF Connect app is acceptable and feasible as self-administered learning for caregivers raising children with FASD. In addition, the FMF Connect app aligns with important reported caregiver values and builds on our previous work evaluating the initial design and functionalities of the app [26]. The sixth phase in our systematic evaluation of the FMF Connect app (Figure 1) is to conduct a larger pilot trial with pre-post quantitative data collection, which is currently ongoing. The results of this pilot trial will provide direction for further refinements to the FMF Connect intervention, measurement tools, and study design methods before the advent of a large-scale RCT. Surprisingly, many mHealth and digital health interventions have been disseminated without clear empirical validation. In our systematic development and evaluation plan, a carefully designed RCT is an important seventh and final phase. This systematic approach is squarely aimed at producing the FMF Connect app as a robust mHealth intervention responsive to the needs of a clinical population that deserves high-quality FASD-informed care.

## Appendix

Multimedia Appendix 1 Semistructured questioning route for the focus group and individual interviews.

## Abbreviations

BT1: first round of beta-testing
BT2: second round of beta-testing
FASD: fetal alcohol spectrum disorders
FMF: Families Moving Forward
mHealth: mobile health
PAE: prenatal alcohol exposure
RCT: randomized controlled trial
