# Supplementary material for: Initial Feasibility of the “Families Moving Forward Connect” Mobile Health Intervention for Caregivers of Children With Fetal Alcohol Spectrum Disorders: Mixed Method Evaluation Within a Systematic User-Centered Design Approach
Source: JMIR Form Res. 2021 Dec 2;5(12):e29687. doi: 10.2196/29687 (PMC8686405; doi:10.2196/29687)
Supplement: Multimedia Appendix 1 [file formative_v5i12e29687_app1.docx]

## Multimedia Appendix 1

Supplemental Table 1: Semi-structured questioning route for focus group and individual interviews

| Topic | Introduction Question | Sample Follow Up Questions/Probes |
| --- | --- | --- |
|  |  |  |
| **Global Impressions & Experience** |  |  |
|  | Tell me about your experience with the FMF Connect App | - What parts worked well for you? - What did you not like about the app? - What would you do differently? |
| **Usage/Engagement** |  |  |
|  | What motivated you to want to try FMF Connect? | - What were barriers that kept you from using the app? - Were there any patterns in how or when you used the app? |
| **Technology** |  |  |
|  | How was using the app from a technology standpoint? | - What bugs did you experience using the app? - Were there any parts of the app that were difficult to use? |
| **Utility** |  |  |
|  | How helpful do you think this app is overall for families raising children with FASD? | - What aspects helped with your child? - Who do you think this app would be most helpful for? |
| **Learning Modules** |  |  |
|  | What do you think about the content in the Learning Modules? | - How was going step by step for you? - What did you think about the exercises? |
| **Videos** |  |  |
|  | What do you think about the videos? | - What did you learn from the videos? - Were any videos too long or wordy? |
| **Family Forum** |  |  |
|  | What was your experience like in the Family Forum? | - What motivated you to use the Forum? - What kept you from using the Forum more? |
| **Dashboard** |  |  |
|  | What did you think about the Dashboard? | - Were any parts of the dashboard difficult to use? |
| **Subtopic: Tip of the Day** |  |  |
|  | What did you think about the Tip of the Day? | - Did you experience any barriers to accessing these? |
| **Subtopic: Behavior Tracking Tool** |  |  |
|  | What do you think about the planned Behavior Tracking Tool? | - Are there any changes you would suggest to make this tool more useful or easier to use? |
| **Library** |  |  |
|  | What do you think about the Library? | - What content interested you the most? - Is there any content you want to see more of in the Library? |
| **Notebook** |  |  |
|  | What was your experience using the Notebook? | - What additions or improvements would you like to see? |
| **Logo/Icon** |  |  |
|  | What did you think about the icon or logo of FMF Connect? | - Did you have any emotional response to the logo/icon? |
